# Deep Learning Based Automatic Ankle Tenosynovitis Quantification from MRI in Patients with Psoriatic Arthritis: A Feasibility Study

**DOI:** 10.3390/diagnostics15121469

**Published:** 2025-06-09

**Authors:** Saeed Arbabi, Vahid Arbabi, Lorenzo Costa, Iris ten Katen, Simon C. Mastbergen, Peter R. Seevinck, Pim A. de Jong, Harrie Weinans, Mylène P. Jansen, Wouter Foppen

**Affiliations:** 1 Image Sciences Institute, University Medical Center Utrecht, 3584 CX Utrecht, The Netherlands; l.costa-2@umcutrecht.nl (L.C.); p.seevinck@umcutrecht.nl (P.R.S.); 2 Department of Orthopedics, University Medical Center Utrecht, 3584 CX Utrecht, The Netherlands; v.arbabi@umcutrecht.nl (V.A.); h.h.weinans@umcutrecht.nl (H.W.); 3 Orthopaedic-Biomechanics Research Group, Department of Mechanical Engineering, Faculty of Engineering, Birjand 561, Iran; 4 Department of Radiology, University Medical Center Utrecht, 3584 CX Utrecht, The Netherlands; 5 Department of Rheumatology & Clinical Immunology, University Medical Center Utrecht, 3584 CX Utrecht, The Netherlands; s.mastbergen@umcutrecht.nl (S.C.M.); m.p.jansen-36@umcutrecht.nl (M.P.J.); 6 MRIguidance B.V., 3584 CX Utrecht, The Netherlands; 7 Department of Biomechanical Engineering, Delft University of Technology (TU Delft), 2628 CD Delft, The Netherlands

**Keywords:** tenosynovitis, MRI, deep learning

## Abstract

**Background/Objectives:** Tenosynovitis is a common feature of psoriatic arthritis (PsA) and is typically assessed using semi-quantitative magnetic resonance imaging (MRI) scoring. However, visual scoring s variability. This study evaluates a fully automated, deep-learning approach for ankle tenosynovitis segmentation and volume-based quantification from MRI in psoriatic arthritis (PsA) patients. **Methods:** We analyzed 364 ankle 3T MRI scans from 71 PsA patients. Four tenosynovitis pathologies were manually scored and used to create ground truth segmentations through a human–machine workflow. For each pathology, 30 annotated scans were used to train a deep-learning segmentation model based on the nnUNet framework, and 20 scans were used for testing, ensuring patient-level disjoint sets. Model performance was evaluated using Dice scores. Volumetric pathology measurements from test scans were compared to radiologist scores using Spearman correlation. Additionally, 218 serial MRI pairs were assessed to analyze the relationship between changes in pathology volume and changes in visual scores. **Results:** The segmentation model achieved promising performance on the test set, with mean Dice scores ranging from 0.84 to 0.92. Pathology volumes correlated with visual scores across all test MRIs (Spearman ρ = 0.52–0.62). Volume-based quantification captured changes in inflammation over time and identified subtle progression not reflected in semi-quantitative scores. **Conclusions:** Our automated segmentation tool enables fast and accurate quantification of ankle tenosynovitis in PsA patients. It may enhance sensitivity to disease progression and complement visual scoring through continuous, volume-based metrics.

## 1. Introduction

Tenosynovitis, characterized by the inflammation of the tendon’s synovial sheath, is a pivotal aspect in psoriatic arthritis (PsA) [1]. Its early detection and monitoring are important for initiating timely treatment and preventing joint damage, thereby improving the patient’s quality of life [2,3,4,5].

Imaging evaluation of tenosynovitis has advantages over clinical examinations of PsA and can reflect prodromal phase of PsA [1,6]. The Outcome Measures in Rheumatology (OMERACT) group developed the PsA MRI Scoring System (PsAMRIS), which includes tenosynovitis among other features and uses a semi-quantitative scoring scheme [7,8]. Despite the establishment of PsAMRIS, its discrete nature complicates careful monitoring of often subtle changes in disease activity [9,10]. Moreover, manually interpreting images is time-intensive and relies on the individual judgment of the radiologist [3,11,12,13]. A study by Glinatsi et al. validating the OMERACT PsAMRIS for the hand and foot in a randomized, placebo-controlled trial reported poor baseline interreader reliability for tenosynovitis in the foot (Intraclass Correlation Coefficient (ICC) 0.25–0.44), while good reliability was observed for other PsAMRIS features (ICC 0.72–0.96) [8]. Jin et al. demonstrate how automated segmentation can improve consistency [14].

Quantifying inflammatory features using volumetric and signal intensity-based metrics may offer a more continuous, objective alternative to discrete visual scoring. This approach could be more sensitive to variations when assessing disease activity and response to therapy over time.

In this study, we assess the feasibility of a fully automatic method for segmentation and quantification of MRI tenosynovitis in the foot. We hypothesize that a fully automated segmentation pipeline can serve as a reliable precursor to volume-based quantification of tenosynovitis, offering more sensitive monitoring than visual scoring.

Previous studies have attempted automatic segmentation and quantification of inflammatory pathologies on MRI images. Haj-Mirzaian et al. summarized the feasibility of computer-assisted quantification of MRI inflammatory arthritis pathologies as the responsiveness of scoring methods are limited by their discreet nature and that those systems can be insensitive to early inflammatory changes [3]. They expect that artificial intelligence (AI)-driven approaches, such as ones based on deep learning (DL) algorithms may overcome these limitations [15,16]. Moreover, Momtazmanesh et al. surveyed the used of AI in rheumatoid arthritis and found no studies using DL-based method for automatic segmentation of inflammatory pathologies [17,18]. Schlereth et al. used deep learning for the classification of erosion, synovitis and osteitis in hand MRI of patients with inflammatory arthritis [19]. Aizenberg et al. investigated the feasibility of automatic quantification of tenosynovitis on MRI of the wrist in patients with early arthritis. They used an atlas-based method for segmentation of tendons, extracted the ROIs around tendons and applied fuzzy clustering to identify voxel intensities inside ROI that can be associated with inflammation [20,21,22]. More recently, Hepburn et al. introduced a human–machine workflow for semi-automatic segmentation and quantification of inflammation load in spondyloarthritis [11]. To the best of our knowledge, no previous studies evaluated the feasibility of DL-based, fully automatic segmenting of tenosynovitis from ankle MRI in inflammatory arthritis.

In our study, we assess the feasibility of a fully automatic method for segmentation and quantification of tenosynovitis on MRI. This method relies on automatic, DL-based segmentation of tendons in the ankle instead of being reliant on manual expert pathology segmentation for ground truth generation, which is a very time-consuming task.

## 2. Materials and Methods

This is a retrospective study to determine the feasibility of automatic segmentation and quantification of ankle tenosynovitis. The quantification is done by automatic segmentation of tenosynovitis and its regions of interest.

### 2.1. Data

Serial bilateral (left and right separately) ankle MRI data from 71 patients with PsA, collected across multiple centers as part of the TOFA-PREDICT study (EudraCT Number 2017-003900-28), were utilized. All the patients included in this study provided written consent and the study was approved by the Medical Research Ethics Committee in Utrecht, Netherlands (MREC reference number: NL63439.041.17). Despite being multi-center, the used images had been acquired from the same MRI machine manufacturer with approximately the same parameters. We note that most images were acquired using Philips Ingenia 3T scanners (Philips Healthcare, Best, The Netherlands) across different sites. One scan acquired with a Siemens 1.5T scanner was excluded from analysis to maintain consistency in field strength and acquisition protocol. The specifics of the study have been described in prior publication [23].

A total of 364 3T T1-weighted Proton Density Spectral Adiabatic Inversion Recovery (PD SPAIR) ankle MR images (coming from the 71 patients, each with multiple timepoints and bilateral images) were analyzed for scoring and quantification purposes. Details on the MRI parameters are reported in Table S1 of the Supplementary Material.

Quantification of tenosynovitis around 4 tendon regions, namely the tibialis posterior tendon, flexor digitorum longus tendon, flexor halluces longus tendon, peroneal longus/brevis tendon, was the topic of this study.

Two musculoskeletal radiologists (WF, IK) conducted scoring for tenosynovitis around the abovementioned four specific tendon regions, following the guidelines of PsAMRIS, which were adapted by the study team for assessment of tenosynovitis around the ankle joint [7,8]. Tenosynovitis was assessed using semiquantitative assessment of fluid within the tendon sheaths (0: none, 1: <1/2 tendon thickness, 2: ≥1/2 tendon thickness, 3: ≥1 tendon thickness). In cases of disagreement regarding the tenosynovitis scores, the differences were resolved through follow-up consensus readings.

**Figure 1:**
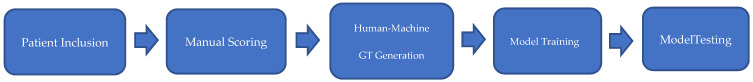
Workflow of the study.

Diagram of the workflow used in this study is as shown in Figure 1.

### 2.2. Ground Truth (GT)

**Figure 2:**
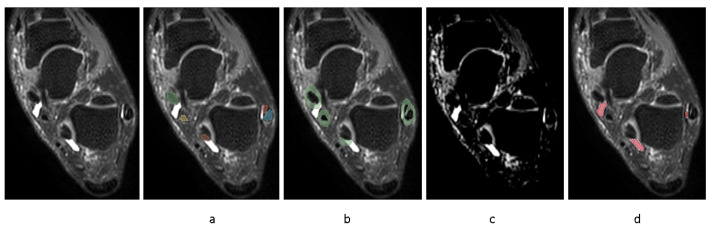
Human–machine workflow of creating ground truth pathology segmentation dataset. (**a**–**d**): (**a**): tendons segmented (colors), (**b**): ROIs around tendons defined (green), (**c**): clustering highlights regions (lighter regions), (**d**): highlighted regions inside ROIs selected (red).

In order to automate the segmentation of tenosynovitis pathologies and validating this process, a dataset of ground truth tenosynovitis segmentation was required. Tenosynovitis ground truth segmentation as shown in Figure 2 was performed in a human–machine workflow to speed up the generation of dataset and to increase the consistency across readers. In the human–machine workflow, a radiologist (WF) supervised an image processing pipeline that generates initial pathology segmentations. These segmentations were then corrected by an MD researcher with 5 years of experience in orthopedics imaging (LC) and approved by radiologist (WF).

The details of the human–machine workflow are as follows: In the first step, as foot tenosynovitis pathologies are around any of 4 tendon regions, these 4 structures were segmented. As peroneal tenosynovitis occurs around both peroneal longus and peroneal brevis tendons, both these structures were segmented. This step is shown in Figure 2a. An MD researcher with 5 years of experience in orthopedics imaging (LC) performed manual segmentations of tendons on a set of 50 MRIs utilizing the 3D Slicer software, version 5.0.1. All 50 tendon segmentations were then reviewed by one musculoskeletal radiologist (WF).

In the majority of cases, the initial segmentations generated by the pipeline were visually plausible and required only minor boundary adjustments or removal of false positives. In fewer cases (−20%), more substantial edits were needed to better capture the extent of inflammation. The human–machine workflow significantly reduced annotation time while ensuring consistency through expert oversight.

Those 50 MRIs (from different patients) were used for segmentation training/testing, while the remaining scans were used for correlation and longitudinal analysis. In order to automate this first step, an nnU-Net [24,25] was trained on 30 full segmentations and tested on 20 full segmentations. nnUNet is a segmentation framework based on U-Net framework [26] that automatically configures the hyperparameters based on the fingerprint of the dataset. The segmentation of tendons is helpful as well in quantification of tenosynovitis, as according to PsAMRIS, tenosynovitis is scored based on inflammation thickness proportionate to tendon thickness.

In step 2, ROIs around the segmented tendons were automatically extracted, as depicted in Figure 2b.

In step 3, fuzzy c-means clustering was applied to the image voxels with the assumption of two clusters (brighter/darker voxels). Then, voxel intensities surpassing the higher cluster center value inside ROI underwent a threshold optimization for probability of those voxels belonging to the cluster. The illustrative result of this clustering method is encapsulated in Figure 2c. The threshold values that yielded the highest correlation of pathology volume with the radiologists’ semi-quantitative pathology scores were taken for further consideration by musculoskeletal radiologist (WF). The radiologist reviewed potential threshold levels on a selection of 10 MRIs from training set, representing various degrees of severity, to identify a probability threshold that most accurately captures the presence of pathology. We applied the same threshold across all four tendon regions, as the signal characteristics of tenosynovitis in these ROIs were visually consistent across images acquired with the same MRI protocol. Importantly, all resulting segmentations were reviewed and, if needed, corrected by an expert reader, ensuring validity regardless of the initial threshold. This ensured that the final annotations used for model training were consistent and accurate. While more advanced, adaptive thresholding techniques may improve automation further, our fixed-threshold approach provided sufficient accuracy for the purposes of this feasibility study.

In step 4, using the chosen threshold value in previous step, inflammatory voxels were automatically segmented and retained if situated within the disease-specific ROI from step 2 or emanated from within this defined area. This is shown in Figure 2d.

Finally, segmentations resulting from step 4 underwent a thorough review of 50 images and any necessary adjustments by MD researcher with 5 years of experience in orthopedics imaging (LC), instructed by a musculoskeletal radiologist (WF). The segmentations were validated and approved by the radiologist (WF).

### 2.3. Data Portions

At the end of the workflow, for each tenosynovitis pathology, a dataset of 50 expert-approved segmentation was thus ready for automation. Thirty segmentations were used for training the segmentation model, and twenty were used for testing the model. To evaluate the generalizability of our segmentation model, we employed a 2-fold cross-validation approach. Specifically, our dataset of 50 labeled segmentations was divided into two folds. For each fold, we used 30 segmentations for training and 20 segmentations for testing. This ensured that all segmentations were used in both training and testing at least once. Each segmentation was included in the testing set once at most, allowing us to obtain a mean test result and assess the model’s performance across different subsets of the data. Data in training and test sets were from different patients to avoid information leak.

### 2.4. Model Training

NnU-Net is inherently structured to compensate for the absence of testing data, employing a 5-fold cross-validation approach as its standard protocol [27]. Given that we had access to an independent testing set within the same dataset, we bypassed this default feature and conducted training using the entirety of the training data. We allocated a distinct dataset for each pathology, processing each one separately. The semi-automatically corrected segmentations were employed to train a deep learning nnU-Net network with 3D full resolution configuration for 1000 epochs using a Dice similarity coefficient with Cross Entropy loss. To enhance the network’s resilience to the various MRI artifacts present in the dataset, the TorchIO data augmentation library [28] was utilized to add noise, ghosting effect or bias field inhomogeneity to images and create augmented image-segmentation pairs. The nnU-Net framework automatically integrates optimal pre-processing and architectural strategies by analyzing characteristics of the given dataset, such as modality, spacing and dimensions of the scans [29].

We selected nnU-Net as our segmentation framework due to its demonstrated state-of-the-art performance across diverse medical image segmentation tasks and its automatic configuration of preprocessing, architecture and training parameters. In our feasibility context with a limited dataset, nnU-Net provided a robust and reproducible pipeline without the need for extensive manual tuning. Although alternative models such as attention-based U-Nets or transformer architectures may offer improvements in specific cases, these were not explored here due to the scope and focus of the study. A recent comparative analysis found that nnU-Net outperformed attention U-Nets and Res-U-Nets across tasks like tumor and polyp segmentation, particularly in recall and Dice score [30].

The code and models used in the study are available at https://github.com/sarbabi/TenosSeg accessed 1 April 2025.

### 2.5. Experiments

Evaluation of automated against semi-automated pathology segmentation.

To assess the efficacy of the trained nnU-Net models, we used them to predict the segmentation of the test scans and compared them with the semi-automatically generated ground truth segmentations available. The training and test sets were selected in a way that covers different scores of tenosynovitis. The test set did not have any patients in common with the training set to avoid information leak. For quantitative evaluation of performance, we employed the Dice coefficient. This indicates how well the trained model can segment the pathology compared to the expert.

### 2.6. Evaluation of Automated Tendon Segmentation

The performance of automated segmentation of tendon regions directly affects the segmentation of ROIs. So, in this part, we evaluate the tendon segmentation model’s performance. The training and tests set were selected randomly from images. The model’s accuracy was benchmarked against the 20 manually segmented MRIs, employing the Dice similarity coefficient as a metric for comparison.

Evaluating human–machine workflow compared to manual method for pathology ground truth segmentations.

To evaluate the consistency and accuracy of the radiologist-instructed pathology segmentations using human–machine workflow, a comparison was made between fully manual segmentations and human–machine workflow. A radiologist (WF) and a radiologist-instructed MD researcher (LC) each performed manual segmentations of tibialis posterior tenosynovitis on 10 randomly selected images from different severities of disease on two separate occasions, one month apart to minimize recall bias. In addition, they each conducted a single segmentation session where they refined machine-generated initial pathology segmentations. The aim of this setup was to assess whether the human–machine workflow might influence the consistency and accuracy of the segmentations as compared to the traditional manual approach. The intra- and inter-observer agreement were calculated using Dice similarity coefficient.

Evaluating correlations of pathology volumes with scores.

Spearman rank correlations between volumes extracted from automatic segmentation of pathologies and the visual scores by radiologists were calculated, to indicate the level of alignment between human qualitative scores and machine quantifications.

### 2.7. Comparing the Sensitivity of Quantifications to Change in Tenosynovitis Score

In order to provide insight into the sensitivity of our methods to detect changes over time in comparison to semi-quantitative scores, the change of inflammation score and volume were illustrated from baseline to week 16, and from week 16 to week 52, in patients where the radiologists unanimously agree on the baseline score. The disease progress, in terms of volume and score on 218 pairs of serial MRIs in 71, patients was shown.

## 3. Results

The MRI parameters for the used PD SPAIR sequence of different centers are reported in Table S1 of Supplementary Materials. A total of 71 patients evaluated using 3T MRI (all centers except five) were included in this study. All included patients were evaluated on MRI scanners from the same manufacturer and using approximately the same parameters for the PD SPAIR sequence. The distribution of scores of radiologists is as depicted in Table S2 of Supplementary Materials.

Performance of the tendon segmentation model.

**Figure 3:**
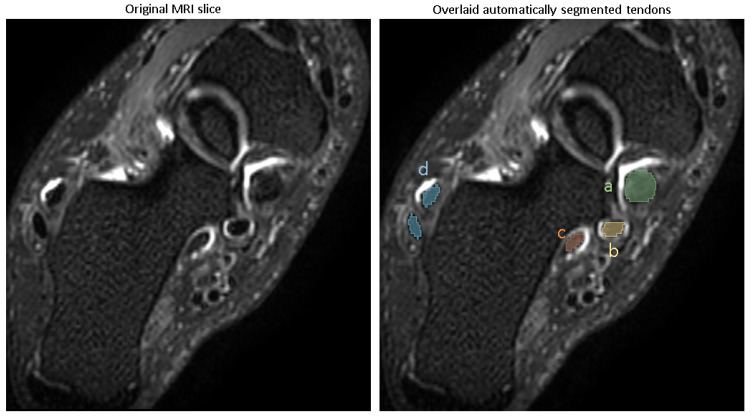
Automatic segmentation of tendons. a: Tibialis posterior tendon, b: flexor digitorum tendon, c: flexor halluces longus tendon, d: peroneal longus/brevis tendon.

A representative segmentation of four tendon regions is demonstrated in Figure 3.

Evaluating the model’s performance with the Dice coefficient on a test set of 20 images disjointed from the training set on the patient level (Table 1), showed that the model achieved a Dice score of (mean ± SD) (0.94 ± 0.01) for the tibialis posterior tendon, (0.93 ± 0.02) for the flexor digitorum longus, (0.91 ± 0.02) for the flexor halluces longus tendon and (0.95 ± 0.01) for the peroneal longus/brevis tendon, reflecting excellent segmentation accuracy.

### 3.1. Automating Pathology Segmentation

**Figure 4:**
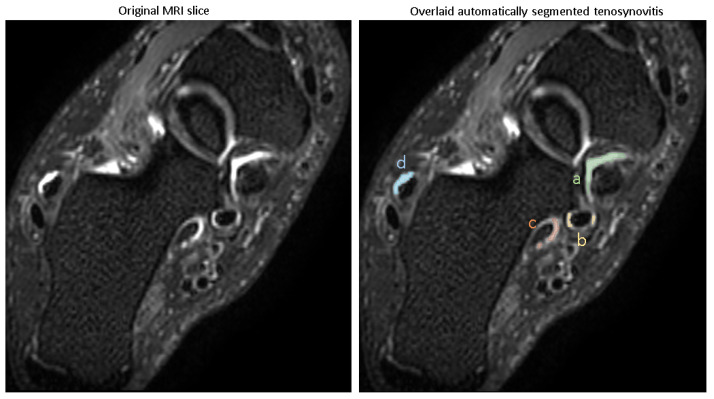
Example image overlaid with automatic segmentation of 4 tenosynovitis pathologies. (a) Tibialis posterior tenosynovitis (score: 3), (b) flexor digitorum tenosynovitis (score: 1), (c) flexor halluces longus tenosynovitis (score: 1), (d) peroneal longus/brevis tenosynovitis (score: 1).

The visual examples of the results of the automatic pathology segmentations for an image are depicted in Figure 4.

The trained models were used for predicting pathologies in the test set, and the Dice scores reported the similarity of the predicted segmentation with the ground truth segmentation. The accuracy of segmentation in terms of Dice score (mean ± SD) is shown in Table 2, with all pathologies showing mean Dice scores of >0.8.

These Dice scores indicate that the models are capable of reliably segmenting tenosynovitis pathologies in the ankle across different regions, with high overlap compared to expert-validated reference segmentations.

### 3.2. Evaluation of Human–Machine Workflow Compared with Manual Segmentation

**Figure 5:**
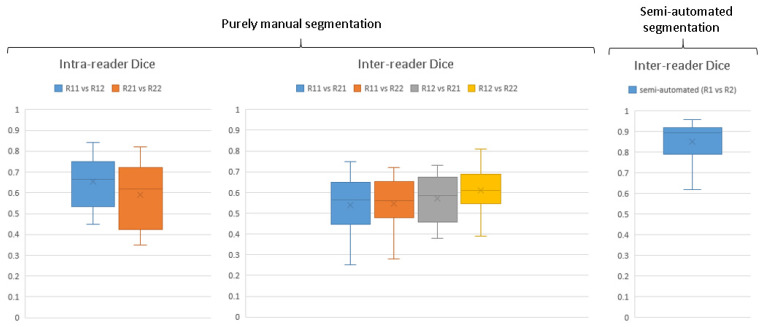
Fully manual and semi-automatic (human–machine) tibialis posterior tenosynovitis segmentation performance. Rij denotes reader i in j-th repetition.

The ground truth segmentations for automating tenosynovitis segmentation were generated using a human–machine workflow. Figure 5 illustrates the enhancement in ground truth pathology segmentation accuracy achieved with the aid of the human–machine workflow, quantified using Dice coefficients. For entirely manual segmentations carried out independently by two observers, the inter-reader agreement in terms of Dice scores varied widely, ranging from 0.25 to 0.81. The median Dice score for repeated segmentations by the same observer (intra-reader agreement) was recorded at 0.66 and 0.62, while the median for segmentations between different observers (inter-observer agreement) ranged from 0.56 to 0.61.

Upon the implementation of the human–machine collaborative workflow, there was a noticeable improvement in the median inter-observer agreement Dice scores, rising to 0.89. This shows a significantly higher agreement between readers for correcting segmentations provided by algorithm than fully manual segmentation of pathology and thereby a need for less refinement by the radiologist in dataset generation.

### 3.3. Threshold Optimization Step

Figure S1 shows the results of the threshold optimization process for tibialis posterior tenosynovitis. As we see, with threshold values between around 0.67 and 0.84, the correlation between pathology volume and radiologist scores is stronger. The radiologist further reviewed this range of thresholds for 10 images stratified from different severities of pathology and based on a threshold value of 0.7 being chosen for tibialis posterior. The same threshold was used for segmenting tenosynovitis in all four regions.

### 3.4. Assessing Correlation Between Scores and Volumes

Evaluating Spearman rank correlation between scores and pathology volume in each ROI for all patients shows ρ = 0.62 for tibialis posterior, 0.58 for flexor digitorum longus, 0.52 for flexor halluces longus and 0.59 for peroneal longus/brevis, with *p*-values < 1 × 10^−16^, showing a moderate to strong correlation. While moderate, these correlations suggest that continuous volume-based measurements align meaningfully with expert semi-quantitative assessments and may provide a more sensitive alternative for longitudinal monitoring.

### 3.5. Assessing Inflammation Dynamics Through Volume Feature and Score Variations

**Figure 6:**
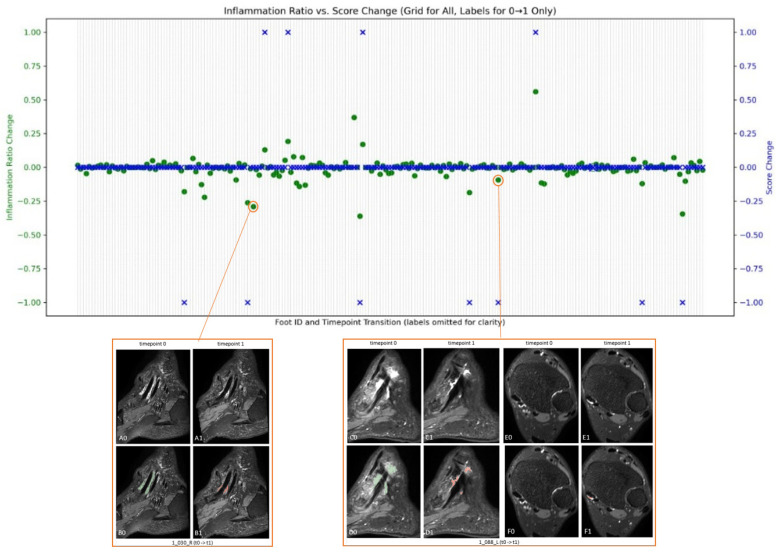
The changes in volume along with scores in all patients. Blue crosses indicate score changes over time as scored by a radiologist, while green dots indicate volume changes as automatically segmented. This figure also illustrates one image that shows negative change in the volume of pathology between the two time-points while the score does not change. Also another example where score negatively changes, but the change in volume is not big.

The disease progress in terms of volume and score on 218 pairs of serial MRIs were evaluated for 71 patients. Figure 6 shows the changes in tibialis posterior tenosynovitis volume and tibialis posterior tenosynovitis scores over time.

For patient 1_030_R, depicted in the bottom left of Figure 6, although at both times the pathology is scored as 1 (score change is 0), the quantification suggests a decrease in inflammation volume from baseline to week 16. In the same way, for patient 1_088_L, illustrated in the bottom right, the baseline score is 3, while at week 16, it is scored as 2. While the overlaid automatic pathology segmentation also depicts this change in C0 to C1, it also indicates a possible increase in pathology shown in E0 to E1.

In summary, the model achieved high segmentation performance across all target pathologies, with Dice scores exceeding 0.8. Volume measurements derived from these segmentations showed statistically significant moderate correlations with radiologist-assigned visual scores. In the longitudinal analysis, volume changes were observed in cases with and without changes in visual score, suggesting the potential sensitivity of continuous quantification for tracking disease activity. These findings support the feasibility of using deep learning-based volume quantification to complement conventional scoring methods.

## 4. Discussion

This research assesses the feasibility of the application of AI in evaluation and monitoring of ankle tenosynovitis through MRI. The findings in this study indicate that the developed automated system could augment the traditional semi-quantitative scoring with more change-sensitive measurements of tenosynovitis, offering quicker results with reduced dependence on human judgment. This is consistent with existing research, which shows AI’s potential to address the challenges of subjective assessments by reducing human error and bias in image analysis and offering continuous-scale measurements.

Although the automated segmentation models achieved Dice scores above 0.8 and moderate to strong correlations with visual scoring, these metrics do not imply perfect agreement or direct clinical interchangeability. The model-derived volume measures offer a continuous, objective view of inflammation burden that complements—but does not replace—expert assessment.

The implementation of a collaborative workflow integrating human expertise and machine capabilities has markedly improved the uniformity with which various observers pinpoint specific regions of interest in pathology images. This improvement implies that the synergy between human knowledge and machine generation of initial segmentations can substantially enhance the precision of data annotations. Our findings demonstrate that the human–machine workflow not only expedited dataset creation but also significantly improved consistency across readers. Compared to fully manual segmentation, which showed a wide range of inter-observer agreement (Dice: 0.56–0.61), the collaborative review of model-generated segmentations led to a much higher agreement (Dice: 0.89). We believe that the model’s initial segmentation served as a stable reference, reducing subjective variability and ensuring more reproducible delineation of pathology—particularly in cases with subtle or borderline inflammation.

The automatic segmentation and volume quantification of tenosynovitis showed moderate to strong correlation with visual scores of tenosynovitis. This shows that our automatic quantification tool aligns favorably with human expert scores, while providing continuous data, which enables more accurate evaluation of changes over time. While Spearman correlations between visual scores and segmented pathology volumes ranged from 0.52 to 0.62, this moderate strength is consistent with expectations given the fundamental differences in data representation. Visual scores are discrete, semi-quantitative values with known inter-reader variability, whereas automated volume quantification provides continuous measures of inflammation. These differences inherently limit perfect correlation. However, the observed correlations—combined with longitudinal consistency—suggest that volume-based quantification may serve as a useful complementary tool to visual scoring, with potential to improve sensitivity in monitoring inflammation over time. Further validation is needed to determine its ability to detect subtle or early changes.

Furthermore, the AI model’s ability to detect volume changes in cases where visual scores remain stable suggests potential sensitivity to subtle inflammatory dynamics. However, these volume fluctuations require further validation—such as radiologist re-scoring or clinical outcome studies—to confirm their clinical relevance. Such enhancements complement traditional radiological assessments, reducing the likelihood of overlooked cases. This is especially evident in the case studies where the quantification of inflammation showed changes over time that were not reflected in the tenosynovitis scores. However, the clinical relevance of the subtle abnormalities and changes over time as provided by quantifications needs further studies on minimally detectable changes and minimally clinically important difference.

Our automated platform is capable of performing quick tenosynovitis pathology segmentation and augmenting the scoring with segmentation for each image.

There are limitations to consider regarding this study. The main limitation is the small pool of expert-validated images for training and testing, attributed to the limited availability of expert validations. Future studies would benefit from a larger dataset. Despite this, the method demonstrated encouraging outcomes across 20 validation sets for each pathology and showed minimal signs of overfitting where there was no large unexpected difference between mean Dice scores in the testing and training sets. Differences in mean dice score between the training and testing sets were between 2.4% to 5.6% for different tenosynovitis pathologies. Another limitation arises from the fact that only little heterogeneity is present in our dataset. Although our data come from different sites, the MRIs are from the same MRI machine model and were acquired with very similar parameters. Only site 5, with one patient, had a different machine, which we did not include in analysis, since our inclusion criterion had been 3T MR images. It remains to be investigated how well the method performs on scans from other vendors. Further validation in independent cohorts and across scanner vendors is required to confirm generalizability for clinical deployment.

An additional challenge encountered in this study was the presence of MRI artifacts, including issues with fat suppression and the ghosting effect. However, assessment of fluid within the tendon sheaths, which appear as hyperintense in the fluid-sensitive sequences used, is not hampered by suboptimal fat suppression. In addition, we employed data augmentation strategies to accommodate the image discrepancies caused by these artifacts. The effects of the artifacts are therefore expected to be limited.

A further limitation stems from the two-stage image-scoring process, where radiologists first assess each MRI independently, followed by a consensus meeting in case of differing opinions. Although this method helps standardize evaluations, it might inadvertently lead to an artificially high agreement rate due to a learning effect at the outset. The act of collaborating to reach a consensus could produce a higher agreement rate than in scenarios where consensus is reached only after all MRIs have been reviewed. As a result, the inter-reader agreement rates reported might not accurately reflect the range of variability that might be seen in routine clinical practice or other research environments.

These limitations highlight the need for a meticulous interpretation of the study’s results and identify areas for improvement in future research.

As this was a feasibility study, our work focused on implementing and validating a self-configuring segmentation pipeline using nnU-Net. While nnU-Net provides strong baseline performance with minimal manual tuning, future work could explore alternative architectures—such as attention-based U-Nets or transformer-based models—which may offer enhanced sensitivity to small or ambiguous pathological features. In addition, benchmarking against external segmentation frameworks and commercial or open-source tools would help assess generalizability and identify areas where our pipeline may be improved or adapted for broader clinical use.

Despite these limitations, our results suggest that deep learning-based quantification of tenosynovitis is feasible and potentially valuable as a complementary tool for objective inflammation tracking in research settings. Further validation in independent cohorts and expanded clinical studies is needed to confirm its robustness and applicability.

## 5. Conclusions

In conclusion, this study assessed the feasibility of augmenting tenosynovitis scoring with AI-generated pathology segmentation. The findings from this feasibility research highlight the potential of AI to automatically quantify ankle tenosynovitis on MRI with accurate pathology segmentation masks. The deep learning-based method showed moderate to strong agreement with visual scores and was able to track longitudinal changes in inflammation. While this is promising, further validation on independent datasets, exploration of clinical thresholds, and studies on reader reliability are needed before broader clinical adoption.

## Figures and Tables

**Table 1:** Dice coefficients of automatic segmentation of tendons.

Tendon	Dice Score
Tibialis posterior	0.94 ± 0.01
Flexor digitorum longus	0.93 ± 0.02
Flexor halluces longus	0.91 ± 0.02
Peroneal longus/brevis	0.95 ± 0.01

**Table 2:** Dice scores on test set for different pathologies (mean ± SD).

Pathology	Test Dice
Tibialis posterior tenosynovitis	0.91 ± 0.04
Flexor digitorum tenosynovitis	0.84 ± 0.07
Flexor halluces longus tenosynovitis	0.85 ± 0.06
Peroneal longus/brevis tenosynovitis	0.92 ± 0.04

## Data Availability

The MRI data used in this study were collected as part of the TOFA-PREDICT trial and contain sensitive patient information. Due to ethical and legal restrictions related to patient privacy and the conditions of the informed consent, the data are not publicly available. Access to the data may be requested from the corresponding author, subject to approval by the appropriate institutional review boards and data use agreements.

## Supplementary Materials

The following supporting information can be downloaded at: https://www.mdpi.com/article/10.3390/diagnostics15121469/s1, Table S1. Center inclusions, scanners, MRI parameters. Table S2. The number of images scored with each grade for each pathology. Figure S1. Threshold optimization for pathology extraction.
