# Supplementary material for: Deep Learning Based Automatic Ankle Tenosynovitis Quantification from MRI in Patients with Psoriatic Arthritis: A Feasibility Study
Source: Diagnostics (Basel). 2025 Jun 9;15(12):1469. doi: 10.3390/diagnostics15121469 (PMC12191622; doi:10.3390/diagnostics15121469)
Supplement: Supplementary file 1 [file diagnostics-15-01469-s001.zip › diagnostics-3640687-supplementary.pdf]

Table S1. Center inclusions, scanners, MRI parameters.

Center	# Patients	# Male, # Female	Weight (Min-Max) Avg	Age (Min-Max) Avg	MR Scanner Manufacturer	MR Scanner Model	Echo Time	Repetition Time	Flip Angle	Magnetic Field Strength	Voxel Size
1	45	29,16	(61–99)81.3	(19–70)52.2	Philips	Ingenia	181.8–183.2	1100	90	3	0.41 * 0.41 * 0.8
2	12	6,6	(50.7–110)79.7	(28–70)48.7	Philips	Ingenia	182.8	1100	90	3	0.41 * 0.41 * 0.8
3	5	3,2	(64–140)87.2	(38–59)50.2	Philips	Ingenia	171.8–171.9	1100	90	3	0.41 * 0.41 * 0.8
4	2	1,1	(85–87)86	(49–50)49.5	Philips	Ingenia	182.4	1100	90	3	0.41 * 0.41 * 0.8
5	1	1,0	75	46	Siemens	Aera	77	1100	120	1.5	0.78 * 0.78 * 0.8
6	2	1,1	(69–95)82	(23–58)40.5	Philips	Ingenia	182.8	1100	90	3	0.41 * 0.41 * 0.8
7	1	1,0	108.4	60	Philips	Ingenia	182.3	1100	90	3	0.41 * 0.41 * 0.8
8	4	2,2	(64–99)85.5	(20–55)45	Philips	Ingenia	182.3–182.8	1100	90	3	0.41 * 0.41 * 0.8

Table S2. The number of images scored with each grade for each pathology.

**Visual Score**	**0**	**1**	**2**	**3**
Tibialis posterior tenosynovitis	66	186	93	12
Flexor digitorum tenosynovitis	265	71	20	4
Flexor halluces longus tenosynovitis	182	91	22	74
Peroneal longus/brevis tenosynovitis	217	118	20	1

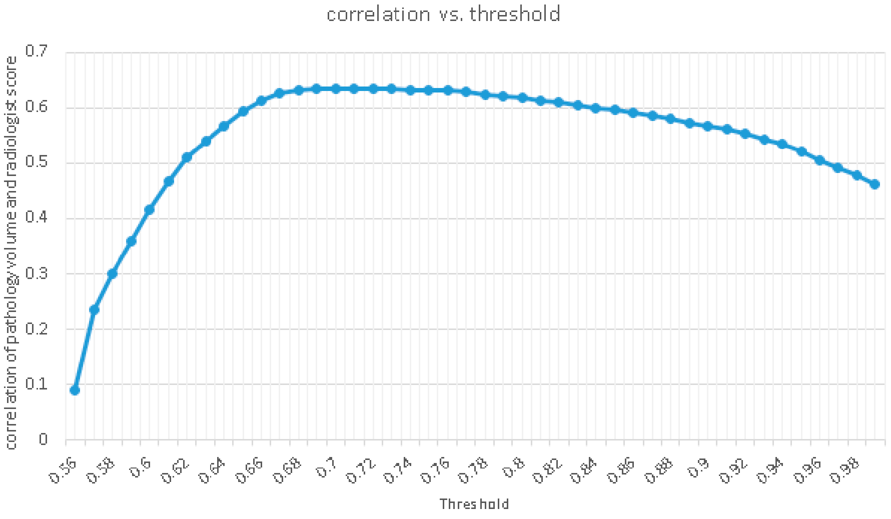

Figure S1. Threshold optimization for pathology extraction.
